# G-protein β3 Subunit Gene 825C/T Polymorphism Is Not Associated with Olanzapine-Induced Weight Gain in Korean Schizophrenic Patients

**DOI:** 10.4306/pi.2009.6.1.39

**Published:** 2009-03-31

**Authors:** Young-Min Park, Young-Cho Chung, Seung-Hwan Lee, Kang-Joon Lee, Hyun Kim, Jung-Eun Choi, Seung-Gul Kang, Min-Soo Lee, Leen Kim, Heon-Jeong Lee

**Affiliations:** 1 Department of Psychiatry, Inje University College of Medicine, Goyang, Korea.; 2 Department of Psychiatry, Korea University College of Medicine, Seoul, Korea.; 3 Division of Brain Korea 21 Biomedical Science, Korea University College of Medicine, Seoul, Korea.

**Keywords:** Olanzapine, Weight, Polymorphism, Schizophrenia

## Abstract

**Objective:**

Weight gain is a possible adverse effect of the use of antipsychotics, and is an important factor for long-term health and treatment compliance. Olanzapine is an atypical antipsychotic known to cause considerable weight gain. A relationship between weight gain and the G protein β3 subunit gene (GNB3) 825C/T polymorphism has been reported. We therefore examined this possible association in a Korean schizophrenic patient group receiving olanzapine treatment.

**Methods:**

Weight and height measurements were obtained prior to starting olanzapine and measured again after long-term treatment. Genotyping for the 825C/T polymorphism was performed using a PCR-based method.

**Results:**

We found that long-term treatment with olanzapine resulted in mean gains in weight and body mass index (BMI) of 5.2 kg and 1.93 kg/m^2^, respectively. There was a no significant difference in the mean body weight change from baseline to the endpoint after olanzapine treatment between the genotype groups (p=0.796). There were also no significant differences in genotype or allele frequencies between the severe weight-gain (more than 10%) and minimal weight-gain (less than 10%) groups (χ^2^=0.037, p=0.98; χ^2^=0.020, p=0.89).

**Conclusion:**

The finding from this study thus does not support a relationship between the GNB3 825C/T polymorphism and weight gain in Korean schizophrenic patients receiving olanzapine treatment.

## Introduction

Weight gain, as a side effect from the use of atypical antipsychotics, has become a serious problem. More than half of those treated with these drugs will gain at least 10% of their initial body weight over the course of therapy, and gains of more than 40% have been reported.^1^ Obesity and weight gain in adulthood are associated with significant health complications such as type II diabetes, coronary heart disease, stroke, gallbladder disease, osteoarthritis, sleep apnea, respiratory problems, and some cancers.^2^ Furthermore, medication-induced weight gain has been associated with a lower quality of life,^3^,^4^ and is a leading barrier to continued compliance with psychiatric medications.^5^-^7^ The dibenzodiazepine-derived drugs, such as clozapine and olanzapine, appear to confer the greatest weight-gain liability. Most of the antipsychotic- induced weight gain occurs during the first 6-8 weeks of treatment and reaches a plateau by the end of the first year of treatment.^8^

The underlying mechanisms by which olanzapine and other second-generation antipsychotics cause weight gain remain unclear; however, there are some pharmacological clues, such as roles for the serotonin (5-HT),^9^ histamine, and adrenergic^10^ receptors. These neurotransmitter receptors are therefore good candidates for an association study of antipsychotic-induced obesity. Wang et al.^11^ reported an association between the G protein β3 825C/T polymorphism and long-term clozapine-induced weight change in a Chinese population. Further to this, Bishop et al.^12^ recently reported a clinically significant relationship between GNB3 genotypes and both negative-symptom responses and weight gain in schizophrenic patients treated with olanzapine. However, the results of that study did not reach statistical significance due to the small sample size.

G proteins are important regulators of the specificity and temporal characteristics of cellular signaling. Heterotrimeric G proteins are composed of three subunits, which dissociate into constituent Gα and Gβγ subunits following receptor activation.^13^ Mutations in the G-protein subunits may therefore contribute to changes in second messenger pathways.^14^ Siffert et al.^15^ detected a singlenucleotide polymorphism (SNP) of 825C/T in exon 10 of the gene encoding the G-protein β3 subunit gene (GNB3). The Gβ3s variant has been associated with increased signal transduction and ion transport in the cell, and with pathophysiological conditions such as hypertension,^15^-^19^ obesity,^20^,^21^ seasonality,^22^ and depression.^23^ In addition, Dishy et al.^24^ reported an association between the GNB3 825C/ T polymorphism and weight gain during pregnancy. A pharmacogenetic study of sibutramine therapy showed the GNB3 825C/T SNP to be a good predictive marker for weight reduction,^25^ although other similar studies failed to demonstrate this association.^17^,^26^,^27^

In light of the previously reported link between the GNB3 825C/T SNP and alterations in body weight regulation,^20^,^28^,^29^ we aimed to investigate the relationship between the GNB3 825C/T SNP and olanzapine-induced weight gain in Korean schizophrenic patients.

## Methods

### Subjects

A total of 104 schizophrenic patients were enrolled from the three collaborating hospitals of Korea University Hospital. All subjects were examined by trained psychiatrists using the Korean version of the Structured Clinical Interview for Diagnostic and Statistical Manual of Mental Disorders, fourth edition (DSM-IV),^30^ leading to a diagnosis based on DSM-IV criteria.^31^ Exclusion criteria included evidence of other psychiatric, medical, or neurological illness; family history of diabetes or eating disorders; and age over 65 or under 18 years. Application of these criteria resulted in the exclusion of 25 patients. Therefore 79 patients were included in this study. All the subjects were ethnic Koreans. Some findings from these subjects have been reported previously.^32^-^34^ Written informed consents were obtained, and the study protocol was approved by the Ethics Committee of the Korea University Hospital.

The subjects were weighed prior to starting olanzapine and again after long-term treatment of at least 3 months. The dosage was adjusted individually according to clinical judgment. The use of drugs other than olanzapine was prohibited. Medications such as antipsychotics, mood stabilizers, and antidepressants were avoided during the study due to their potential effects on weight change; however, we combined the use of benzodiazepines or anticholinergics as needed. No subject had received olanzapine or clozapine prior to the present study. The mean daily dose of olanzapine at the end-point examination was 13.6 mg (SD=5.3 mg).

Other clinical variables that were measured in the study were gender, age, olanzapine treatment duration and dosages, and previous antipsychotics dosages (expressed as chlorpromazine equivalents). Changes in body weight and body mass index (BMI) during the treatment were also calculated.

### Genotyping

Genomic DNA was extracted from leukocytes of the study subjects using a QIAamp Blood Kit (Qiagen, Germany). A polymerase chain reaction (PCR)-based method was used for genotyping of the GNB3-gene 825C/T polymorphism.^15^,^23^

### Statistical analyses

Differences in allele frequencies between cohorts with different body weight changes were evaluated by a chi-square analysis. The association of genotype with weight gains and changes in BMI was assessed by univariate analysis of variance (ANOVA) or Student's t-test. All of the analyses were performed using standard software (SPSS for Windows), and probability values of p<0.05 were considered statistically significant.

## Results

Genotype frequencies in our sample were not deviated from Hardy-Weinberg equilibrium (χ^2^=0.008, p=0.93). The sociodemographics, initial body weight and BMI, olanzapine dosage, previous antipsychotic dosages, and treatment duration did not differ between the genotype groups. There was a no significant difference in the mean body weight change from baseline to the endpoint after olanzapine treatment between the genotype groups (p=0.796). When we divided the subjects into two groups, the T-allele carriers (the TT and CT genotypes) and the T-allele non-carriers (the CC genotype), the mean changes in body weight from baseline to endpoint after olanzapine treatment were still not significantly different (p=0.515). The other clinical variables did not differ significantly between the two groups (Table 1). There were also no significant differences in genotype or allele frequencies between the severe weight-gain (more than 10%) and minimal weightgain (less than 10%) groups (χ^2^=0.037, p=0.98; χ^2^=0.020, p=0.89)(Table 2).

## Discussion

One of candidate genes for an association with obesity is the heterotrimeric G proteins, which are key components of intracellular signal transduction and play a focal role in adipogenesis.^2^,^35^,^36^ A previous study showed that the GNB3 825C/T variant influences lipolysis.^37^ In the heterotrimeric G protein complex, the β subunit of the G protein complex is common for both G_s_ and G_i_ complexes, and is ubiquitously expressed.^38^ Therefore, a functional variation in GNB3 may influence the action of G_s_- as well as G_i_-coupled receptors. The β_1_- and β_2_-adrenoceptors (G_s_-coupled), and β_2a_-adrenoceptors (G_i_-coupled) coexist in human fat cells, where they activate and inhibit lipolysis, respectively.^37^ The 825T allele of the GNB3 gene appears to decrease the production of GNB3 in fat cells and thereby inhibit lipolysis via β_1_-, β_2_-, and β_2a_-adrenoceptor signaling.^37^ From a study of post-pregnancy weight retention, Gutersohn et al.^28^ reported that subjects with a GNB3 825 T/T genotype are at high risk for obesity and post-pregnancy weight retention if they do not exercise regularly. The 825 T allele of the GNB3 gene is associated with an increased BMI across different ethnicities, and apparently represents a "thrifty genotype".^20^,^21^ Several studies,^20^,^21^,^28^,^37^,^39^ have reported a significant relationship between the GNB3 825C/T polymorphism and obesity or fat metabolism. In contrast, Suwazono et al.^40^ reported that the GNB3 825C/T SNP was not a significant factor in being overweight for 2,625 Japanese people. In addition, Tsai et al.^41^ reported that the GNB3 825C/T SNP was not significant in clozapineinduced body-weight change. In the present study, we also did not find a sig-nificant association between GNB 825C/T SNP and olanzapine-induced weight gain, which was consistent with the previous Asian studies.^40^,^41^ It is possible that there are ethnic differences in the relationship between GNB3 825C/T SNP and obesity; however, recently, Ujike et al.^42^ reported the 825T allele of GNB3 was significantly associated with olanzapine-induced weight gain in 164 Japanese schizophrenic patients. Very recently, Souza et al.^43^ investigated the association of the GNB3 and antipsychotic-induced weight gain using meta-analytical techniques. Their analysis of 18,903 subjects showed no significant association although there was a trend associating CC and lower BMI under a fixed model. Therefore, this relationship remains controversial.

The major limitation of this work was that our patients had already received traditional antipsychotics prior to the study, and therefore any tendency to weight gain may have been triggered already. However, we observed that the chlorpromazine-equivalent doses of the previous antipsychotics, baseline body weight, and baseline BMI did not differ between the genotypes. The second issue was that the duration of medication was not the same among our study cohorts, possibly influencing the results. We do not believe, however, that any such effect would be significant, because there was no difference in duration of olanzapine treatment between the genotype groups, and previous studies reported that most weight gain occurred during the first 6-8 weeks of olanzapine therapy^8^ Third, we did not assess and control caloric intake in the subjects (including caloric counts and meal refusals) due to the nature of a long-term study. Finally, the relatively small sample size studied here limits the generality of our findings.

Additional studies using larger sample sizes and better medication control are needed. It will also be necessary to evaluate the possible involvement of an as-yet-uncovered gene(s) that influence susceptibility to olanzapine-induced weight gain as well as the possibility of gene-gene interactions.

## Figures and Tables

**TABLE 1:**
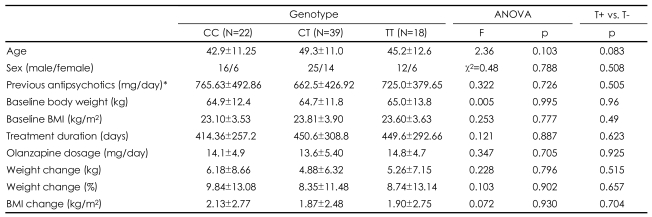
Demographic and clinical variables of 79 schizophrenic patients in the three genotype groups

Values represent mean±SD. ^*^Chlorpromazine equivalents. ANOVA: analysis of variance, BMI: body mass index

**TABLE 2:**
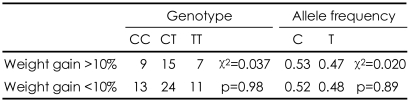
Comparison of the GNB3 genotypes and allele frequencies between higher (>10%) and lower weight gain (<10%)

GNB3: G-proteisn beta subunit gene
